# Safety and Tolerability of an Eye Drop Based on 0.6% Povidone–Iodine Nanoemulsion in Dry Eye Patients

**DOI:** 10.1089/jop.2020.0085

**Published:** 2021-03-04

**Authors:** Giovanni William Oliverio, Rosaria Spinella, Elisa Imelde Postorino, Leandro Inferrera, Emanuela Aragona, Pasquale Aragona

**Affiliations:** ^1^ Department of Biomedical Sciences, Ophthalmology Clinic, University of Messina, Messina, Italy.; ^2^ Department of Ophthalmology, Vita-Salute University, IRCCS San Raffaele Scientific Institute, Milan, Italy.

**Keywords:** povidone–iodine, dry eye disease, microbiota, antiseptic, antibiotic resistance, ocular surface

## Abstract

***Purpose:*** To evaluate safety and tolerability on the ocular surface of an anti-septic formulation containing 0.6% povidone–iodine (0.6% PVI) for a 4 week period.

***Methods:*** An observational, prospective study included 20 mild-moderate dry eye disease (DED) patients who enrolled at the Ocular Surface Disease Unit of the University of Messina, receiving 0.6% PVI eye drops for 28 days, 2 drops twice daily (BID). The assessment included the Ocular Surface Disease Index questionnaire; symptoms score (0 = absent to 3 = severe) for burning, ocular dryness, foreign body sensation, watery eyes, tearing, photophobia, and ocular pain; fluorescein tear break-up time (TBUT); and corneal-conjunctival staining, performed at baseline (T0), after 7 (T7) and 28 (T28). Schirmer *I*-test, corneal endothelial cell count, intraocular pressure, and fundus examination were performed at T0 and T28. The main outcome measures were TBUT and corneal-conjunctival staining as markers of ocular surface homeostasis. For statistical analysis, Student's *T*-test and Wilcoxon test were used as appropriate.

***Results:*** No significant alterations of the safety parameters were found throughout the study. Further, at T28 a significant improvement of burning, ocular dryness, foreign body sensation, and watery eyes (T0 vs. T28 *P* < 0.03) were observed; corneal-conjunctival staining improved at T28 (T0 vs. T28 *P* < 0.0001), and TBUT improved already at T7 (T0 vs. T7 *P* = 0.0008) lasting so till the end of the study. The only adverse event was mild burning at instillation for the first 3 days of treatment in most of the patients.

***Conclusions:*** The treatment with 0.6% PVI was safe and well tolerated in a group of patients with a damaged ocular surface.

## Introduction

Povidone–iodine (PVI) is a commonly used antimicrobial agent, composed by a complex of hydrogen iodide, elemental iodine, and polyvynilpyrrolidone, a synthetic polymer.^1^

The broad spectrum of antimicrobial activity and its efficacy, particularly in relation to resistant micro-organisms, are well documented.^2^

As a result of its broad spectrum of microbicide activity, 5% solution of PVI is routinely used in ophthalmic surgery for the prophylaxis against postsurgical endophthalmitis.^3–5^

A novel ophthalmic eye drops formulation, in suspension, of 0.6% PVI, sodium hyaluronate (SH), and medium-chain triglycerides (MCT) nanoemulsion (0.6% PVI) was manufactured and available on the market.

Basic science research showed that concentrations of PVI solutions <1% present the highest content of bactericidal-free iodine.^6,7^

The 0.6% PVI solution proved to be efficacious in antibacterial prophylaxis for the intravitreal injection procedure, reducing the conjunctival bacterial load and the risk for needle contamination.^8^

Emerging resistance to many commercially available antibiotics, given to PVI in ophthalmic solution, presents an interesting potential as an alternative treatment to prevent infections. A recent experimental, *in vitro* study showed a rapid activity of 0.6% PVI against multi-resistant strains of *Staphylococcus aureus*, *Staphylococcus epidermidis,* and *Pseudomonas aeruginosa*.^9^

Therefore, 0.6% PVI represents a valid prophylactic method of ocular surface antisepsis, as demonstrated by recent studies.^8–10^

Corneal epithelial damage was demonstrated after repeated 5% PVI applications, but more diluted solutions showed a better tolerability.^11^

Considering the growing use of 0.6% PVI, it is important to know not only its effectiveness as an antimicrobial agent but also its tolerability and safety profiles, in particular in subjects with pre-existing alterations of the ocular surface, such as dry eye disease (DED) patients. In fact, its formulation, including substances such as SH, MCT, glycerol, and vitamin E d-alpha-Tocopherol Polyethylene Glycol (vit E TPGS), seems to contains factors that may favor the ocular surface protection.

The aim of this study is to evaluate the safety and tolerability of 0.6% PVI on the ocular surface after a long-term treatment period.

## Methods

This observational, prospective, single-center study involved 20 mild-moderate DED patients (18 females, 2 males; mean age 61 ± 12.3 years) recruited at the Regional Referral Centre for the Ocular Surface Diseases of the Department of Biomedical Sciences, University of Messina, Messina, Italy.

The study was approved by the Institutional Review Board of the University of Messina, protocol number 86/17, and was conducted in accordance with the tenets of the Declaration of Helsinki.

Informed consent was obtained from all participants after the explanation of nature and the possible consequences of the study.

Inclusion criteria were: age between 18 and 80 years old, the presence of qualitative or quantitative alterations of the ocular surface and tear film dysfunction demonstrated by tear film break-up time (TBUT) <10 s, ocular surface fluorescein staining (NEI/Industry workshop score) >3, and Schirmer's *I*-test <8 mm in 5 min.^12,13^ Exclusion criteria were: glaucoma, history of herpes simplex or herpes zoster keratitis, history of corneal ulcer, allergic keratoconjunctivitis, use of contact lenses, and previous ocular treatments within 6 months, including anti-inflammatory and immunosuppressant drugs for the treatment of dry eye. The only treatment allowed was tear substitutes, whose use was stable for at least 3 months before the beginning of the study.

### Treatment performed

All patients were treated with 0.6% PVI eye drops (IODIM; Medivis, Catania, Italy) 2 times daily (BID) for 28 days, and they underwent assessment at baseline (T0), and after 7 (T7) and 28 days (T28). The composition of the 0.6% PVI solution was reported in Table 1.

**Table 1.** Composition of the Povidone–Iodine-Based Sterile, Isotonic Eye Drops

Povidone iodine 0.6%
Vitamin E TPGS
Glycerol
Mineral salts (sodium, potassium, chloride, and citrate)
Medium-chain triglycerides (MCT)
Sodium hyaluronate 0.05% 1.8M D

Vitamin E TPGS, Vitamin E D-alpha-Tocopherol Polyethylene Glycol; 1.8M D, 1.8 Million Daltons.

### Tests performed

- Ocular symptoms were evaluated by means of the Ocular Surface Disease Index (OSDI) questionnaire, a test designed to score the severity of dry eye, administered before the ophthalmologic assessment. The questionnaire consisted of 12 questions about the respondent's past-week experience with ocular symptoms, vision-related functioning, and environmental triggers. This was performed at T0, T7, and T28.^14,15^
- A symptom score questionnaire (from 0 = absent to 3 = severe) for burning, ocular dryness, foreign body sensation, watery eyes, tearing, photophobia, and ocular pain was also obtained at T0, T7, and T28.
- Best-corrected visual acuity was obtained at T0 and T28.
- The Fluo imaging function of Keratograph 5M (K5M; Oculus GmbH, Wetzlar, Germany) was used to study the ocular surface after fluorescein instillation. For TBUT, 1 drop from a fluorescein strip (Bio Glo, Fluorescein sodium ophthalmic strips; HUB Pharmaceuticals, LLC Rancho Cucamonga, CA) wetted with saline solution [Sodio Cloruro 0.9%; S.A.L.F. S.p.A. Laboratorio Farmacologico, Cenate Sotto (BG), Italy] was instilled onto the lower fornix; the patients were invited to blink 3 times to allow an even distribution of the fluorescein, and then they were asked to stare without blinking as long as they could. The time gap between the last complete blink and the appearance of a dark spot on the corneal surface was recorded as the time of tear film resistance (s) and was calculated, for statistical purposes, as the average of 3 consecutive measurements.^13^ The assessment was carried out at T0, T7, and T28.
- Three minutes after the TBUT assessment, the ocular surface fluorescein staining score was obtained according to the scheme proposed by the NEI/Industry workshop.^12^ The epithelial staining was evaluated and photographed, with Keratograph 5M with a score from 0 (absent) to 3 (areas of complete loss of epithelium), for each of the 5 corneal sectors considered, with a total score for corneal staining ranging from 0 to 15 and for conjunctival staining from 0 to 18, since 6 areas were considered (2 nasal, 2 temporal, 1 superior, and 1 inferior). The total score for both corneal and conjunctival stain was added and recorded as 1 single value, indicative of the global epithelial condition, and it was used for statistical purposes. To obtain this value pictures from the cornea and superior, inferior, nasal, and temporal conjunctiva were used. The assessment was performed at T0, T7, and T28. The images obtained were assessed in a masked fashion by 2 experienced observers (P.A. and E.I.P.). An interobserver accordance above 95% was achieved.
- For Schirmer *I*-test, filter paper strips (Test di Schirmer, Alfa Intes, Casoria, Italy) were applied at the junction between the outer and the middle third of the lower lid. The moisturized length of the strip was measured after 5 min (mm/5 min). The test was performed at T0 and T28.

The safety profile was further assessed at T0, T7, and T28 by slit-lamp examination to evaluate the anterior chamber, and lens changes were seen as possible signs of an intraocular inflammatory reaction.

Further, the corneal endothelial cell count, carried out with a Perseus endothelial microscope (CSO, Scandicci, Florence, Italy); intraocular pressure measurement, performed with Oculus Corvis ST (Oculus, Wetzlar, Germany); and fundus examination, carried out with Daytona (Optos, Dunfermline, Scotland, United Kingdom), were performed at T0 and T28.

### Statistical analysis

Population of the study: To identify the numerosity of the population of the study was considered an improvement of 50% for TBUT, based on the reported effect of the SH solutions.^16^ Using an alpha error of 0.05 and a beta error of 80%, the population number obtained was 16; therefore, 20 patients were enrolled to allow a possible dropout. However, no dropouts were reported. The main outcome measures were TBUT and corneal-conjunctival staining.

For statistical analysis, the MedCalc version 12.2.1.0 statistical software for Windows (MedCalc Software, Ostend, Belgium) was used. Data were described as mean ± standard deviation.

The Student's *t*-test for parametric data was applied to assess the significance of differences between baseline and treatment at T7 and T28 data; Wilcoxon signed-rank test was used for nonparametric data. Values from the worst eye at baseline were taken into account for statistical purposes. A *P* value <0.05 was considered statistically significant.

## Results

The clinical parameters evaluated at T0, T7, and T28 are reported in Table 2.

**Table 2.** Clinical Parameters at Baseline and After 7 and 28 Days of Treatment

	Baseline	95% CI	Day 7	95% CI	Day 28	95% CI
Burning	2 ± 0.8	1.75–2.25	1.9 ± 0.8	1.69–2.21	1.2 ± 0.8^a,b^	1.0–1.49
Ocular dryness	2.2 ± 0.8	1.95–2.44	2 ± 0.8	1.79–2.31	1.6 ± 0.8^c,d^	1.28–1.76
Foreign body sensation	1.8 ± 0.4	1.67–1.93	1.7 ± 0.6	1.57–1.92	1.1 ± 0.8^e,f^	0.89–1.41
Watery eyes	0.7 ± 1.0	0.42–1.07	0.7 ± 1.0	0.42–1.07	0.4 ± 0.8^g^	0.16–0.63
Tearing	0.1 ± 0.3	0.00–0.19	0.1 ± 0.3	0.00–0.19	0.1 ± 0.3	0.00–0.19
Photophobia	0.5 ± 0.8	0.25–0.75	0.5 ± 0.8	0.25–0.75	0.4 ± 0.3	0.18–0.65
Ocular pain	0.1 ± 0.3	0.00–0.19	0.1 ± 0.3	0.00–0.19	0.1 ± 0.3	−0.01–0.16
OSDI	19.4 ± 7.5	16.70–21.95	19.6 ± 7	17.30–21.97	18.8 ± 6.1	16.36–21.19
TBUT	2.2 ± 0.7	1.92–2.40	2.65 ± 1.3^h^	2.07–3.02	4 ± 1.5^i,j^	3.42–4.47
Ocular surface staining	3.3 ± 0.5	3.15–3.44	3.3 ± 0.4	3.15–3.44	1.6 ± 0.9^k,l^	1.25–1.85
Schirmer *I*	4.8 ± 4.5	3.18–6.19			4.6 ± 4.1	3.17–6.19
Endothelial cell density	2746.7 ± 306.6	2506.5–2851.2			2789.8 ± 297.5	2551.3–2884.8
Intraocular pressure	15.4 ± 2.1	13.9–16.2			14.9 ± 2.2	13.4–15.7

Data are presented as mean ± standard deviation.

^a^ *P* = 0.0002 (vs. baseline).

^b^ *P* = 0.0005 (vs. day 7).

^c^ *P* = 0.0003 (vs. baseline).

^d^ *P* = 0.006 (vs. day 7).

^e^ *P* = 0.0001 (vs. baseline).

^f^ *P* = 0.0003 (vs. day 7).

^g^ *P* = 0.03 (vs. baseline).

^h^ *P* = 0.0008 (vs. baseline).

^i^ *P* < 0.0001 (vs. baseline).

^j^ *P* < 0.0001 (vs. day 7).

^k^ *P* < 0.0001 (vs. baseline).

^l^ *P* < 0.0001 (vs. day 7).

CI, confidence interval; OSDI, ocular surface disease index; TBUT, tear film break-up time.

No changes were found for visual acuity, slit-lamp exam for anterior chamber and lens, endothelial cell count, intraocular pressure, and fundus exam.

The only adverse event reported by 16 patients (80%) was a mild burning on the instillation, lasting a few minutes. Such symptom disappeared after about 3 days of treatment.

At the end of the treatment, all patients showed a significant improvement of the symptoms evaluated with the scoring system: In particular, statistically significant differences were observed for burning (*P* = 0.0002), ocular dryness (*P* = 0.0003), foreign body sensation (*P* = 0.0001), and watery eye (*P* = 0.03) at the T28 visit with respect to baseline values (Table 2).

The OSDI values were reduced but did not reach a statistically significant difference. The TBUT showed a statistically significant increase starting from day 7 (*P* = 0.0008) and lasting until the end of the treatment (*P* < 0.0001) (Table 2).

**FIG. 1.**
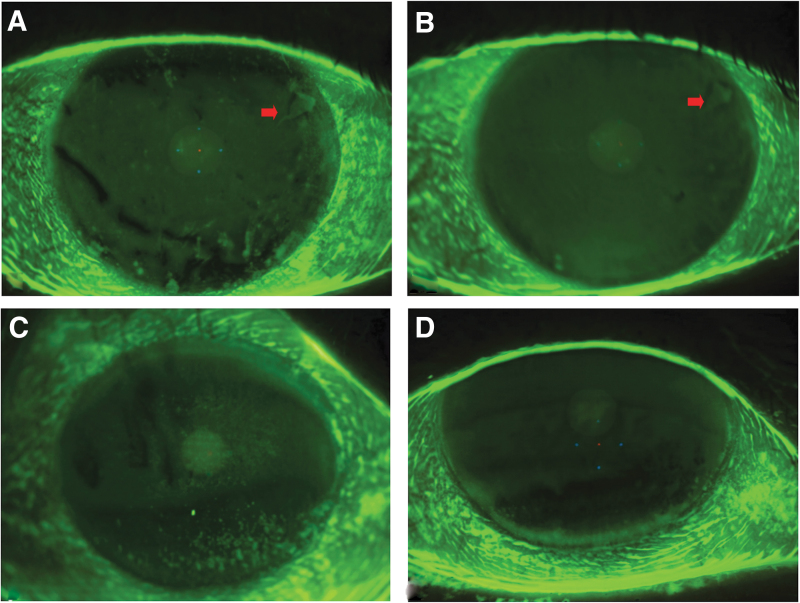
Fluorescein staining of the ocular surface. Please note the evident reduction of corneal staining after 28 days of treatment **(B, D)** versus baseline **(A, C)**. The *arrow* indicates the presence of an alteration of the corneal epithelial basement membrane at baseline **(A)** and after 28 days of treatment **(B)**.

A statistically significant reduction in ocular surface staining was noted at the end of the treatment (*P* < 0.0001 vs. T0) (Figure 1).

Schirmer *I*-test did not show significant changes at the end of the study period versus T0 values.

## Discussion

The resistance to antibiotics is a worldwide growing emergence mostly derived by their inappropriate use; even their topical administration on the conjunctiva may induce changes of the ocular surface microbiota, not only reducing its diversity but also promoting the proliferation of resistant bacterial genera and an alteration of the microbiota/epithelial cell interaction. This can provoke a further shift toward a state of infection and/or of chronic inflammation, secondary to epithelial activation.^17–19^

Antiseptics could represent an alternative or adjuvant treatment for the infections of the ocular surface.^20^ However, repeated and prolonged exposure to antiseptic agents, such as benzalkonium chloride, chlorhexidine, and polyhexamethylene biguanide (PHMB), could potentially induce toxic side effects to the ocular surface cells.^21–25^

Five percent PVI is routinely used in ophthalmic surgery, for the prevention of postoperative infections. PVI solutions release free iodine, which penetrates the microbial membrane, causing death due to the oxidative stress brought on the cells.^26^ Unfortunately, this outcome is also true for eukaryotic cells, and therefore, the ocular surface epithelium could be damaged by the repeated and/or prolonged instillation of PVI eye drops. Studies demonstrated the toxicity on both epithelial and endothelial corneal cells after treatment with povidone–iodine at concentrations higher than 1% and/or treatment longer than 2 min.

Clinical and experimental data showed the toxic effects of topical iodine on the ocular surface epithelium, particularly after repeated exposure.^6,11,27–29^ In fact, the prolonged exposure to 5% PVI of the damaged corneal epithelium might result in keratocyte death and persistent epithelial defects, due to the disruption of the stromal–epithelial interaction with the consequent retarded re-epithelialization.^27^

PVI, in several concentrations, proved to have a virucidal activity documented against herpes simplex virus, adenovirus, as well as coronavirus, responsible for the recent epidemic outbreaks such as the severe acute respiratory syndrome coronavirus (SARS-CoV) and the middle-east respiratory syndrome coronavirus (MERS-CoV).^10,30–32^

Compared with 5% PVI, more diluted PVI solutions showed a better safety profile in an experimental study carried out on rabbits. After the application of 10 drops of PVI with different dilutions (5%, 2.5%, 1%, and 0.5%), the corneas were studied at the slit lamp after 30 min. The application of the 2.5% solution resulted in lower damage than the 5% solution, whereas no damage at all was demonstrated for the 1% and 0.5% solutions.^11^ This effect may also derive from the very low pH of the more concentrated solutions: In fact, the more diluted are the solutions, the higher is the pH, closer to that of tears.^6^

Saedon et al. reported the detrimental effects of repeated intravitreal injection procedures for macular disorders on symptoms of dry eye.^33^ It was supposed that this effect may be related to the high concentrations of PVI used (5% for 3 min) that can affect the health of the ocular surface epithelium. Other studies proved the efficacy of 0.6% PVI solution in reducing the bacterial load with the presurgical treatment in intravitreal injections, and also in the treatment of infectious keratitis and conjunctivitis.^8,10^

Further, an *in vitro* study proved the antimicrobial efficacy of a more diluted formulation, containing 0.6% PVI, which showed a faster bactericidal activity compared with the traditional 5% PVI solution. The 0.6% PVI revealed *in vitro* antimicrobial activity against *S. epidermidis*, *S. aureus*, *P. aeruginosa*, and, to a lesser extent, *Candida* spp.^9^

The greater bactericidal efficacy of 0.6% PVI, compared with more concentrated solutions, has been related to the increased availability of free iodine, which is an active antimicrobial component.^6^

However, the possible positive effects of PVI instillation might be thwarted by pre-existing ocular surface alterations, such as those present in DED patients, who could be more sensitive to any toxic effect determined by PVI treatment.

To explain the effect of 0.6% PVI treatment on DED, it could be hypothesized that ocular surface microbiota might be involved in it.

In fact, numerous evidences confirmed its role, stating that the ocular surface microbiota, bacterial biofilm, and bacterial lipases may influence the occurrence of DED and other ocular surface disorders.^34–36^ The microbiota plays a fundamental role in determining ocular surface immunological homeostasis. In DED patients, the changes in microbiota components may form the basis of the reduced tolerance mechanisms and determine the induction of both innate and adaptive immunity, characteristic of the chronic inflammatory process typical of DED.^37,38^ This can contribute to ocular surface damage characterized by corneal-conjunctival squamous metaplasia with goblet cell reduction. In moderate-to-severe DED patients, a greater bacterial presence was described,^39–41^ with a consequent loss of the ocular surface immunologic tolerance. In fact, goblet cells, through the production of transforming growth factor-β (TGF-β), participate in the immune tolerance process, typical of the healthy ocular surface condition, by selecting tolerogenic dendritic cells, which are able to stimulate the homing of regulatory T cells (T-regs) on the ocular surface.^42–48^

The use of antiseptic substances, such as PVI, can abolish germs overgrowth that is responsible for the induction of an inflammatory status, without inducing resistance-related problems. So, in DED patients, the antiseptic effect of PVI can decrease the bacterial population, resulting in a reduction of the inflammatory stimulus on the ocular surface epithelial cells.^40^

The formulation of 0.6% PVI tested in this study is characterized by a high dilution with an increased pH, with respect to less diluted formulations; further, the presence of other components may favor good epithelial tolerability.

The tested 0.6% PVI formulation contains an SH molecule; it extensively described the great water retention ability and non-Newtonian viscoelastic behavior of SH that increase the epithelium wettability.^49–57^ However, the SH molecule, present in the formulation, has a high molecular weight (1.8 M of Daltons) but a very low concentration (0.05%). For this reason, it does not have the rheological characteristics of proper artificial tears formulations. Nevertheless, SH is effective in reducing the irritating effects of free iodine on the ocular surface epithelia in a PVI formulation.

Other important components are MCT, which may improve the tear film stability ameliorating the lipid layer.^58–60^

Glycerol is a compatible solute substance that, together with the mineral salts, contributes toward equilibrating the tear film osmolarity, thus reducing the hyperosmolar damage on the epithelial cells.^61^

Another important component is the hydrophilic vit E TPGS, present as a surfactant in the 0.6% PVI formulation; it is a potent antioxidant agent and is responsible for the protection of eukaryotic epithelial cells against the oxidation induced by free iodine through reactive oxygen species derived from the alteration of cell membrane components, such as lipids and proteins, and nucleic acids.^26,62–66^

The aim of our study was to evaluate the safety and tolerability of 0.6% PVI treatment with an already damaged ocular surface, such as mild-moderate DED patients. Patients enrolled were of both DED types, namely evaporative and aqueous deficient. The results showed that some dry eye-specific symptoms, such as burning, ocular dryness, foreign body sensation, and watery eye, improved at the end of the treatment. However, these improvements did not bring significant changes in OSDI values. It is possible to state that the improvement of the symptoms was linked to the statistically significant amelioration of TBUT and epithelial damage, as demonstrated by corneal-conjunctival stains. No serious adverse events were reported during the study, apart from a mild-moderate burning sensation on the instillation in some patients that disappeared after about 3 days from the beginning of the treatment.

As to the safety aspects, it was possible to demonstrate the absence of alterations regarding visual acuity, corneal endothelial cells, lens transparency, intraocular pressure, and fundus. Since mild-moderate dry eye patients usually do not show changes in visual acuity but rather a possible reduction of contrast sensitivity, as a consequence of tear film quality alteration,^67^ we considered TBUT as a safety marker, indirectly representing possible changes in vision quality.

Anterior chamber reaction, demonstrated by the presence of cells and proteins, can be an indicator of anterior segment suffering with iris and ciliary body involvement.

Lens transparency is often altered by topic treatments such as those with some NSAIDs.

The evaluation of corneal endothelial cells carried out with the specular microscope is important to evaluate possible damage to these cells, described for higher concentrations of PVI.

Intraocular pressure and fundus exams are usual safety parameters studied in several trials.

We preferred to study the TBUT after fluorescein staining of the ocular surface, because in this way it was possible to obtain the evaluation of the whole corneal surface, differently from the non-invasive BUT (NIK-BUT) function of the Keratograph 5M, which is based on the reflection of Placido rings projected on the corneal surface that do not cover the entire corneal area.

Therefore, it is possible to conclude that 0.6% PVI appears to be safe on the ocular surface. Further, its use could improve signs and symptoms in DED patients. It can be hypothesized that 0.6% PVI acting on ocular surface microbiota may rebalance the anomalous bacterial overgrowth typical of DED, thus promoting the prevalence of immune tolerance on inflammatory mechanisms. Moreover, for the aforementioned reasons, the use of 0.6% PVI could be considered in the antiseptic treatment of repeated para-surgical procedures, such as intravitreal therapies.

The limitations of the study are that this is an observational, prospective study carried out without a control group. However, our main interest was to evaluate the possible toxic effects brought by the 0.6% PVI formulation on the ocular surface and we chose mild-moderate DED patients as an ideal population to be investigated with regard to this aspect. Nevertheless, further studies specifically addressing the possible use of 0.6% PVI as a complementary treatment for dry eye are warranted. Further studies addressing the ocular surface microbiota changes, the effect on visual function, and the modified corneal-conjunctival structure, evaluated by *in vivo* confocal microscopy, are necessary to better elucidate the possible mechanisms underlying the ocular surface modifications induced by 0.6% PVI treatment.
